# Costs of hospitalization with respiratory syncytial virus illness among children aged <5 years and the financial impact on households in Bangladesh, 2010

**DOI:** 10.7189/jogh.07.010412

**Published:** 2017-06

**Authors:** Mejbah Uddin Bhuiyan, Stephen P Luby, Nadia Ishrat Alamgir, Nusrat Homaira, Katharine Sturm–Ramirez, Emily S. Gurley, Jaynal Abedin, Rashid Uz Zaman, ASM Alamgir, Mahmudur Rahman, Ismael R. Ortega–Sanchez, Eduardo Azziz–Baumgartner

**Affiliations:** 1 International Centre for Diarrheal Disease Research, Dhaka, Bangladesh; 2 School of Paediatrics and Child Health, University of Western Australia, Perth, Australia; 3 Centres for Disease Control and Prevention, Atlanta, Georgia, USA; 4 James P Grant School of Public Health, BRAC Institute of Global Health, BRAC University, Dhaka, Bangladesh; 5 Discipline of Paediatrics, School of Women’s and Children’s Health, University of New South Wales, Randwick, Australia; 6 Oxford Policy Management, Oxford, UK; 7 Institute of Epidemiology, Disease Control and Research, Dhanka, Bangladesh

## Abstract

**Background:**

Respiratory syncytial virus (RSV) is the leading cause of acute respiratory illness in young children and results in significant economic burden. There is no vaccine to prevent RSV illness but a number of vaccines are in development. We conducted this study to estimate the costs of severe RSV illness requiring hospitalization among children <5 years and associated financial impact on households in Bangladesh. Data of this study could be useful for RSV vaccine development and also the value of various preventive strategies, including use of an RSV vaccine in children if one becomes available.

**Methods:**

From May through October 2010, children aged <5 years with laboratory–confirmed RSV were identified from a sentinel influenza program database at four tertiary hospitals. Research assistants visited case–patients’ homes after hospital discharge and administered a structured questionnaire to record direct medical costs (physician consultation fee, costs for hospital bed, medicines and diagnostic tests); non–medical costs (costs for food, lodging and transportation); indirect costs (caregivers’ productivity loss), and coping strategies used by families to pay for treatment. We used WHO–Choice estimates for routine health care service costs. We added direct, indirect and health care service costs to calculate cost–per–episode of severe RSV illness. We used Monte Carlo simulation to estimate annual economic burden for severe RSV illness.

**Findings:**

We interviewed caregivers of 39 persons hospitalized for RSV illness. The median direct cost for hospitalization was US$ 62 (interquartile range [IQR] = 43–101), indirect cost was US$ 19 (IQR = 11–29) and total cost was US$ 94 (IQR = 67–127). The median out–of–pocket cost was 24% of monthly household income of affected families (US$ 143), and >50% families borrowed money to meet treatment cost. We estimated that the median direct cost of RSV–associated hospitalization in children aged <5 years in Bangladesh was US$ 10 million (IQR: US$ 7–16 million), the median indirect cost was US$ 3.0 million (IQR: 2–5 million) in 2010.

**Conclusion:**

RSV–associated hospitalization among children aged <5 years represents a substantial economic burden in Bangladesh. Affected families frequently incurred considerable out of pocket and indirect costs for treatment that resulted in financial hardship.

Globally, an estimated 34 million new episodes of respiratory syncytial virus (RSV)–associated acute respiratory infection occurred among children aged <5 years in 2005, of which 2.8–4.3 million episodes required hospitalization [1]. The incidence of RSV–associated ARI among young children in low–income countries is believed to be more than twice than that of the high–income countries (59 vs 24/1000 per year) [1]. RSV has been reported as an important viral contributor to the pneumonia burden among young children in both rural and urban settings in Bangladesh [2,3] and is the leading viral pathogen for hospitalization of children aged <5 years with severe acute respiratory illness [4].

The annual direct health care cost of hospitalization because of RSV illness among young children in high–income countries was estimated at US$ 24–394 million [5–8]. For those countries, the annual economic burden of illness provides an estimate of government expenditures due to the illness that can inform implementation of appropriate cost–effective prevention strategies. In contrast, in most low–income countries, including Bangladesh, where the majority of health care cost is paid out–of–pocket in the absence of public health insurance systems [9], an annual economic estimate of a particular illness reflects the financial impact of the illness on households. There is little information about the economic burden of RSV illness in low–income countries such as Bangladesh where the RSV–associated disease burden is high [1]. In 2007, a cost of illness study conducted at the largest pediatric hospital in Bangladesh estimated that the mean medical cost for a hospitalized child with pneumonia was US$ 94 per illness episode and nearly two–thirds of families in this study had to spend more than half of their monthly expenditure for treatment [10]. Although the previous cost study lacks cost estimates for viral respiratory illness, the findings suggested that costs for hospitalization with RSV pneumonia could be catastrophic for affected families because of existing high RSV burden.

While there is currently no licensed vaccines to protect against RSV, a number of vaccines are in development [11]. Palivizumab, an intramuscular monoclonal antibody is recommended for young infants with high risk conditions including congenital heart disease, congenital lung disease or prematurity to prevent severe RSV infection, but is not currently cost-effective for use in Bangladesh because of its high cost (US$ 6000 per child per season) [12,13]. Using 2010 cost data from hospitalized persons with laboratory–confirmed viral illnesses, we aim to analyze the costs associated with severe RSV hospitalization among children aged <5 years in Bangladesh. Specifically, the objective is to estimate out–of–pocket, health care system and indirect costs from the societal, provider and family perspectives as well as the financial hardship imposed on families of sick children. These data may be used to evaluate the value of various preventive strategies, including use of an RSV vaccine in children if one becomes available.

## METHODS

### Surveillance settings

We conducted this study in the catchment areas of ongoing sentinel childhood respiratory illness surveillance at three public and one private hospital in four districts in Bangladesh [14]. As part of the surveillance activity, two days per month, a surveillance physician in each hospital enrolled all children aged <5 years residing in the catchment area who were hospitalized with any two of the following symptoms during admission: reported or measured fever, cough or difficulty breathing. Nasal and throat swabs were collected from enrolled children. The specimens were kept in liquid nitrogen and transported to icddr,b virology laboratory fortnightly to test for the presence of RSV using real time reverse transcription polymerase chain reaction (rRT–PCR) [15].

### Study population and enrolling case–patients

We conducted this study during the influenza epidemic period, May through October, as the primary objective of the original study was to estimate economic burden of influenza in Bangladesh. The seasonality of RSV in Bangladesh is well defined but we anticipated finding laboratory–confirmed RSV cases at the sentinel hospitals during those months as well [2,3,16]. During May through October, 2010, investigators identified all children aged <5 years from the sentinel childhood respiratory illness surveillance database who had laboratory–confirmed RSV infection and defined them as case–patients. Research assistants telephoned each case–patient’s parents, informed them that their child had laboratory–confirmed viral respiratory illness and obtained verbal consent for a home visit to administer a standard questionnaire about the cost associated with the hospitalization. Then, research assistants visited each case–patient’s home and if any parents were unavailable during the first visit, field research assistants made two more visits to that dwelling in an attempt to collect cost information.

### Data collection

We used the same data collection tool to capture direct and indirect costs, previously used for influenza cost estimation in this setting [17]. Research assistants interviewed case–patients’ parents using a structured questionnaire to obtain socio–economic information such as the highest household educational attainment, monthly household income, accumulated household assets, source of water supply, cooking fuel and latrine type, self–reported costs incurred at other health care service facilities prior to visiting the sentinel hospital for that illness episode, cost associated with hospitalization at sentinel hospitals, impact of treatment cost on household economy and the coping strategies that each household used to pay treatment cost [17]. In cases when parents reported about borrowing money as coping strategy, research assistants collected data on the lender and interest rate. The interest rate was standardized into per annum rate.

### Cost components

#### Direct costs

We categorized the direct cost into medical and non–medical costs [18]. Cost incurred for hospital registration fees, bed rent, medicine, laboratory tests, and informal payments (comprised payments to hospital support staff for arranging beds for admitted children on the floor of the ward, obtaining oxygen cylinders for children). for the illness were considered as medical costs. The respondent identified which medications and laboratory tests they received from the hospital free of charge (hospital subsidized cost) and which they purchased out–of–pocket. Research assistants collected cost information for hospital registration fees and bed rent from hospital receipts. Research assistants also collected self–reported non–medical cost such as caregivers’ food, lodging and transportation costs. We used WHO–Choice estimates for routine health care service costs per bed–day for any hospitalized patients at tertiary level hospitals in Bangladesh [19] to calculate costs for that illness episode from providers’ perspectives. WHO–choice estimates the routine health care service cost that includes health–care providers and support staffs salary, capital cost and patients’ food cost. WHO–Choice data excludes cost of any drugs and laboratory tests, whether subsidized or paid out–of–pocket, which were collected from the patients’ caregiver.

#### Indirect costs

We collected self–reported daily wage and number of work days lost by each family caregiver due to the illness event. For employed caregivers, loss of work days excluded weekends or leave days. For caregivers who were daily–wage earners or homemakers (mothers), we considered any day lost for caring for sick children as a lost work day. We did not assign an indirect cost for days of restricted activity such as half a day of work or when a caregiver reported that the illness episode did not interrupt their regular activity.

### Cost calculation

We calculated prescribed medicine and laboratory test costs using price lists collected from local drug stores and laboratories during the study period. We calculated hospital subsidies cost and out–of–pocket cost for medicine and laboratory tests. We then added medical, non–medical and health care service cost to estimate the direct cost–per–episode. We estimated indirect cost (ie, work time lost) of caregivers using the human capital approach [20]. We assigned monetary value to the time lost by caregivers by multiplying missed work days by self–reported daily wages. To calculate the value of work time lost by unemployed caregivers such as mothers and grandparents, we used the minimum wage in Bangladesh during 2010 [ie, Taka 100/d or US$ 1.4/d] [21] and multiplied this rate by the number of work days missed. This approach was previously used by other researchers in measuring economic burden of illness [22]. For multiple caregivers of the hospitalized child, we calculated work time loss of each caregiver and then summed these to estimate the total indirect cost–per–episode for each hospitalized child. We added cost incurred at other health care facilities to total direct and indirect cost–per–episode to obtain the total cost–per–episode of RSV–associated hospitalization. Initially, we estimated each cost in Bangladeshi currency (Taka) and then converted our estimates into US$ according to the average exchange rate during the time of data collection (US$ 1 = Taka 70) [23]. We multiplied the incidence rate for RSV hospitalization (8.3/1000 children year) from a recent study in Bangladesh [16] by the estimated national population of children <5 years during 2010 to estimate the annual number of children aged <5 years with RSV–associated hospitalization in Bangladesh in 2010 [24]. We then multiplied the median cost of an episode for RSV hospitalization by the estimated number of RSV–associated hospitalizations per year to obtain the annual economic burden for RSV–associated hospitalization among children aged <5 years in Bangladesh in 2010. We used a Monte Carlo simulation to resample from empirical distribution of our cost components 1000 times to generate a 95% confidence interval of the estimates [25,26].

### Principal component analysis

We performed a principal component analysis using information about household assets (eg, table, chair, radio, television, and refrigerator) and access to household services (eg, electricity, source of water, fuel and latrine type) to construct a wealth index [27]. We divided the households into two groups on the basis of their median principal component score. Families below the median were categorized as poor and those who had median scores at or above the median were categorized as wealthier.

### Ethical consideration

We obtained written informed consent from the parents of hospitalized child. The study protocol was reviewed and approved by an icddr,b institutional review committee.

## RESULTS

### Characteristics of study respondents

During May through October 2010, 47 hospitalized children aged <5 years with laboratory–confirmed RSV illness were identified from the surveillance database. Research assistants interviewed parents of 39 (83%) case–patients and were unable to reach the remaining eight (17%) after multiple visits to their dwellings. The monthly household income of participating households was US$ 143 (interquartile range [IQR] = 114–257). The mean duration between case–patients’ hospital discharge and their parents’ interview was 26 days (range = 8–47 days). The median age of the 39 case–patients was 4 months (IQR = 2–7 months) (**Table 1**). Most recruited children (82%, 32/39) had already sought health care before reaching the study hospitals; 41% (13/32) visited doctors with a Bachelors of Medicine and Surgery, 25% (8/32) visited other health facilities, 22% (7/32) visited village doctors, 16% (5/32) visited homeopath doctors and 9% (3/32) visited pharmacies. The median out–of–pocket cost incurred for treatment at other health care providers before reaching the sentinel hospitals was US$ 3.6 (IQR = US$ 1.5–7.2).

**Table 1:** Characteristics of children hospitalized with respiratory syncytial virus (RSV)–associated illnesses and their families, by hospital type, Bangladesh, May–October, 2010

Characteristics	Public hospital	Private hospital	Total
Number of case–patients	25	14	39
Male (%)	18 (72)	9 (64)	27 (69)
Age of case–patients in months:				
≤6 (%)	17 (68)	11(79)	28 (72)
7–12 (%)	6 (16)	2 (14)	8 (20)
13–59 (%)	2 (8)	1 (7)	3 (8)
Median (IQR)	5 (3–7)	3.5(2–6)	4 (2–7)
Monthly household income, median (IQR) US$	143 (100–214)	201 (129–343)	143 (114–257)
Number of household members, median (IQR)	5 (4–7)	6 (4–9)	5 (4–7)
Days from symptom onset to hospital admission, median (IQR)*	4 (3–5)	2 (1–4)	3 (2–5)
Days hospitalized, median (IQR)	5 (3–6)	4.5 (4–5)	5 (3–6)

*Comparisons are between public hospital and private hospital; *P* = 0.01.

### Costs of RSV hospitalization

#### Direct costs

The median direct cost of RSV hospitalization was US$ 62 (IQR = US$ 43–101). The median direct cost in the private hospital was higher than in the public hospitals (US$ 90 vs US$ 48) (*P* = 0.0038) (**Table 2**). Nearly two–thirds (16/25) of case–patients in public hospitals received at least one medicine and/or diagnostic test from the hospital free of charge whereas none of 14 case–patients in the private hospital received any hospital subsidized medicine or diagnostic test. The remaining nine (36%) case–patients in public hospitals and all case–patients in private hospitals paid 100% of medicine and diagnostic costs out–of–pocket. The cost of medicine, on average, constituted 60% (range: 11–92%) of the total direct cost of hospitalization.

**Table 2:** Direct cost–per–episode (in US$) of respiratory syncytial virus (RSV) hospitalization in four hospitals, Bangladesh, May–October 2010

Parameter	Public hospitals		Private hospital		Total	
**N (%)**	**Median (IQR)**	**N (%)**	**Median (IQR)**	**N (%)**	**Median (IQR)**
Medicine cost	25 (100)	21(15–25)	14 (100)	20 (12–27)	39 (100)	22 (15–27)
Diagnostic cost	9 (36)	5(1.5–7.5)	11 (79)	2.6 (2.5–4.0)	20 (50%)	2.7 (2.5–6.6)
Transportation cost^†^	23 (92)	4.2 (2.6–7.3)	14 (100)	8.9 (6.4–17)	37 (95)	6.4 (3.0–11)
Healthcare service cost	25 (100)	23 (14–27)	14 (100)	20 (18–23)	39 (100)	23 (14–27)
Other costs**^‡^**	25 (100)	1.7 (0.6–4.1)	13 (93)	33 (26–37)	38 (97)	3.7 (0.8–27)
Total direct cost/episode*	25	48 (36–64)	14	90 (71–112)	39	62 (43–101)

*Comparisons are between public hospital and private hospital, *P* = 0.0038.

†Transportation costs included round trip cost of case–patient from home to hospital and care–givers travel cost for hospital visit.

**‡**Other costs included cost for hospital registration, informal payment, food, hospital bed and lodging of caregiver.

#### Indirect costs

During the illness, children obtained care from an average of three family caregivers (range: 1–6). Mothers were invariably the primary caregiver (39/39, 100%) with grandmothers also providing some care (21/39, 54%). Caregivers lost a median of 12 days of productivity (IQR = 8–17 days) and a median of US$1.4 (IQR = US$ 1.4–1.6) loss per day during the illness episodes. The median indirect cost of RSV hospitalization was US$ 19 (IQR = US$ 11–29).

#### Total cost

We estimated that the median total cost per episode of RSV hospitalization was US$ 94 (IQR = 67–127) and that direct cost constituted, on average, 67% (95% CI = 62–72) of this total cost.

### Financial impact on affected families

The median out–of–pocket cost for hospitalization with laboratory–confirmed RSV represented 24% of the monthly household income among participating families, 32% among the poorer families and 17% among wealthier families (**Table 3**). None of the families reported having health insurance to cover the treatment cost. More than 50% (20/39) of the families borrowed money to pay for their child’s treatment (**Table 3**). Of 20 families who obtained loans, 13 (65%) obtained interest–free loans from relatives and seven (35%) obtained loans from community lenders at annual interest rates of 50–120%. Four (31%) of the 13 who obtained interest free loans had repaid them whereas none of the seven families who took loans with interest had repaid the loan a month after hospital discharge. Fourteen (70%) of the poorer families and 6 (32%) wealthier families decreased their monthly expenditures on food because of costs incurred during illness.

**Table 3:** Per–capita income per month of case–patient's family (in US$), out–of–pocket costs per episode of RSV hospitalization as a percentage of monthly household income and the coping strategies, Bangladesh, May–October 2010

Parameter	Household category	
**Poorer, N = 20**	**Wealthier, N = 19**
Per capita income per month, median (IQR)	23 (17–25)	38 (29–64)
Out–of–pocket costs as percentage of monthly income, median (IQR)	32 (19–53)	17 (12–27)
Coping strategy:		
Received contribution from relatives, n (%)	3 (15)	2 (11)
Borrowed money, n (%)	13 (65)	7 (37)

IQR – interquartile range

### National cost

We multiplied the rate of RSV hospitalization in children aged <5 years (8.3/10 000 children year) [16], by the census population of children aged <5 years in Bangladesh during 2010 and estimated that approximately 160 500 children aged <5 years were hospitalized with RSV illness in Bangladesh in 2010. We estimated that the median direct cost of RSV–associated hospitalization in children aged <5 years in Bangladesh was US$ 10 million (IQR: US$ 7–16 million), the median indirect cost was US$ 3.0 million (IQR: 2–5 million) in 2010.

## DISCUSSION

RSV is the leading viral cause of acute lower respiratory infections in children, particularly in children younger than 5 years and 99% of RSV–associated deaths occur in low–income countries [1]. RSV vaccines are in development but there is little information about the cost of RSV illness to inform cost–benefit models of RSV vaccination programs. Our data illustrates that RSV–associated hospitalization among children aged <5 years represents a substantial economic burden in Bangladesh and families caring for children with severe RSV illness frequently incur substantive out of pocket and indirect costs that result in financial hardship, particularly among the poorer. Our estimated cost of an episode of severe RSV illness (US$ 94) was similar to a previously published cost estimate for hospitalized pneumonia among children in Bangladesh [10]. We estimated the annual direct and indirect economic burden for RSV–associated hospitalization in Bangladesh; however, if non–hospitalized disease were included the amount might be twice as high because only 51% of children aged <5 years with severe acute respiratory illness are brought to hospitals for evaluation [4,28].

Our data showed that the health care costs associated with RSV hospitalization were primarily paid out–of–pocket, irrespective of type of health facility, public or private. The out–of–pocket costs were as high as one–third of the monthly income of poor families excluding the substantial work time lost for caregivers. The high out–of–pocket expenditures for health care service was somewhat expected as there is no insurance scheme to cover health care costs in Bangladesh, similar to many low–income countries [29,30] and previous cost studies in Bangladesh also had similar findings [10,17]. We found that the direct cost for RSV hospitalization in private hospitals was higher than in public hospitals although the median days of hospitalization in public and private hospitals were similar. Hospital registration, food for patients, and hospital bed charges are subsidized in public hospitals but paid by families in private hospitals. In addition, none of the patients in private hospitals received any hospital supported medicine and/or laboratory tests, which resulted in higher out–of–pocket costs when compared with those in public hospitals.

We found that affected families frequently obtained loans, either interest free from relatives or at usurious rates from local money lenders to meet treatment cost. Similar coping strategies were previously documented in other resource–poor settings to pay for health care costs of ill family members [10,31]. In Bangladesh, the annual interest rate for loans obtained from the informal sectors, such as local money–lenders, is around 180–240% and is much higher than from commercial banks where the rates are ~ 10–13% per annum [32]. Nevertheless, people in rural settings rely on the informal sector to obtain loans because such loans are available quickly and the majority of these poor people are ineligible to get loans from formal sectors as they do not own adequate assets to guarantee their ability to repay the loan [32]. Families in our study who obtained loans from local money lenders were also unable to repay their loan nearly a month after hospital discharge. Loans obtained from the informal sector at extremely high interest rates for RSV treatment could result in long–term debits and may propel families into vicious cycles of poverty [33–35].

A study on out–of–pocket expenditure for health care services in neighboring India suggested the impact of health care payments on families food and children’s education purchasing capacity can be substantial [36]. The parents of this study also mentioned reduction of monthly food expenditure as the foremost impact of treatment cost for their child's illness. These data also highlight how an episode of severe RSV illness that resulted in hospitalization could have an indirect impact on the food availability in affected households, which is concerning for a country like Bangladesh where nearly half of the children aged <5 years are malnourished [37].

Our study had several limitations. We identified RSV–cases from an ongoing surveillance for respiratory illness which seemed to use less sensitive case–definition for RSV illness and thus we likely missed cases of RSV–illness. We estimated the cost of an episode of RSV hospitalization using data from only 39 laboratory–confirmed cases. A more sensitive case–definition might have resulted in the enrollment of patients with a different presentation, treatment profile, and cost of illness. Cases were identified from four rural hospitals and our cost–estimates may not be generalizable to urban areas where 25% of the population lives. Although the study sample size was relatively small and cost estimates largely represented rural population, these cost estimates were similar to previously published pneumonia hospitalization costs at other sites in Bangladesh [10], supporting the representativeness of our cost estimation. Our cost estimates could be underestimates for several reasons. We collected expenditure data from study respondents retrospectively. Some of the respondents were interviewed after a month of hospital discharge and may have forgotten some expenses incurred during illness. In order to improve recall, we went through all billing records, prescriptions and discharge reports of hospitals and also cross–checked claimed medicine cost with local drug stores. To ensure the representativeness of drug prices, research assistants collected the brand name of the drug used by the child during the illness. Because the price of drug of a specific brand is likely to be fixed in most pharmacies in Bangladesh (as set by the producing company), these estimates are likely reliable. We did not count days with restricted activity as it would over–report the productivity loss. To calculate the work time lost by caregivers, we used wage rates self–reported by caregivers which may not have been generalizable. However, the monthly household income of the study families (US$ 143) was similar to the monthly income of a typical rural household in Bangladesh (US$ 138) [38], suggesting that our data might be representative of the whole country. Our estimates of annual direct and indirect cost of RSV–associated hospitalization at the national level was based on an incidence estimate from a single year and RSV burden has been found to vary substantially from year to year in Bangladesh [16]. However, a recent paper on RSV hospitalization from 2010–2014 in Bangladesh showed that RSV hospitalization rate was the highest in 2010 than other years [16], suggesting that the estimated cost represented the economic burden of a season with high RSV activity in Bangladesh.

Severe RSV illness requiring hospitalization of young children seemed to result in a significant annual economic burden in Bangladesh during 2010. Most of the hospitalization costs were paid out–of–pocket and often led families to incur loans. In the absence of government–sponsored health insurance, community–based health insurance programs could help to reduce the household–level economic impact of childhood hospitalization for severe respiratory infection [39]. Cost data for RSV hospitalization from low–income settings, where the disease burden is highest, could be used as a baseline to evaluate the cost–benefit of ongoing efforts to develop affordable RSV vaccines for resource poor settings and also to evaluate the effect of non–pharmaceutical preventive interventions, such as hand washing which have been found effective in reducing RSV transmission [40].

## References

[1] Nair H, Nokes DJ, Gessner BD, Dherani M, Madhi SA, Singleton RJ (2010). Global burden of acute lower respiratory infections due to respiratory syncytial virus in young children: a systematic review and meta-analysis.. Lancet.

[2] Hasan K, Jolly P, Marquis G, Roy E, Podder G, Alam K (2006). Viral etiology of pneumonia in a cohort of newborns till 24 months of age in Rural Mirzapur, Bangladesh.. Scand J Infect Dis.

[3] Homaira N, Luby SP, Petri WA, Vainionpaa R, Rahman M, Hossain K (2012). Incidence of respiratory virus-associated pneumonia in urban poor young children of Dhaka, Bangladesh, 2009-2011.. PLoS One.

[4] Nasreen S, Luby SP, Brooks WA, Homaira N, Al Mamun A, Bhuiyan MU (2014). Population-based incidence of severe acute respiratory virus infections among children Aged <5 Years in Rural Bangladesh, June-October 2010.. PLoS One.

[5] Paramore LC, Ciuryla V, Ciesla G, Liu L (2004). Economic impact of respiratory syncytial virus-related illness in the US: an analysis of national databases.. Pharmacoeconomics.

[6] Stang P, Brandenburg N, Carter B (2001). The economic burden of respiratory syncytial virus-associated bronchiolitis hospitalizations.. Arch Pediatr Adolesc Med.

[7] Ranmuthugala G, Brown L, Lidbury BA (2011). Respiratory syncytial virus–the unrecognised cause of health and economic burden among young children in Australia.. Commun Dis Intell Q Rep.

[8] Deshpande SA, Northern V (2003). The clinical and health economic burden of respiratory syncytial virus disease among children under 2 years of age in a defined geographical area.. Arch Dis Child.

[9] Munsur AM, Atia A, Koffi AK, Kawahara K (2009). Household out-of-pocket expenditures on health care in Bangladesh according to principal component analysis (PCA).. Biosci Trends.

[10] Alamgir NI, Naheed A, Luby SP (2010). Coping strategies for financial burdens in families with childhood pneumonia in Bangladesh.. BMC Public Health.

[11] Haynes LM (2013). Progress and challenges in RSV prophylaxis and vaccine development.. J Infect Dis.

[12] Geskey JM, Thomas NJ, Brummel GL (2007). Palivizumab: a review of its use in the protection of high risk infants against respiratory syncytial virus (RSV).. Biologics.

[13] Resch B, Sommer C, Nuijten MJ, Seidinger S, Walter E, Schoellbauer V (2012). Cost-effectiveness of palivizumab for respiratory syncytial virus infection in high-risk children, based on long-term epidemiologic data from Austria.. Pediatr Infect Dis J.

[14] Azziz-Baumgartner E, Alamgir AS, Rahman M, Homaira N, Sohel BM, Sharker MA (2012). Incidence of influenza-like illness and severe acute respiratory infection during three influenza seasons in Bangladesh, 2008-2010.. Bull World Health Organ.

[15] Mentel R, Wegner U, Bruns R, Gürtler L (2003). Real-time PCR to improve the diagnosis of respiratory syncytial virus infection.. J Med Microbiol.

[16] Homaira N, Luby SP, Hossain K, Islam K, Ahmed M, Rahman M (2016). Respiratory viruses associated hospitalization among children aged <5 years in Bangladesh: 2010-2014.. PLoS One.

[17] Bhuiyan MU, Luby SP, Alamgir NI, Homaira N, Mamun AA, Khan JA (2014). Economic burden of influenza-associated hospitalizations and outpatient visits in Bangladesh during 2010.. Influenza Other Respir Viruses.

[18] Segel JE. Cost-of-Illness Studies-A Primer. 2006. Available: http://rti.org/pubs/COI_Primer.pdf. Accessed 15 December 2011.

[19] World Health Organization. Choosing Interventions that are Cost Effective (WHO-CHOICE): country-specific unit costs. Available: http://www.who.int/choice/country/country_specific/en/index.html. Accessed: 20 June 2012.

[20] Koopmanschap MA, Rutten FF (1996). A practical guide for calculating indirect costs of disease.. Pharmacoeconomics.

[21] Government of the People’s Republic of Bangladesh. activities of Ministry of Labour and Employment for two years (2009-2010). 2010. Available: www.mole.gov.bd. Accessed: 21 January 2011.

[22] Shepard DS, Undurraga EA, Lees RS, Halasa Y, Lum LC, Ng CW (2012). Use of multiple data sources to estimate the economic cost of dengue illness in Malaysia.. Am J Trop Med Hyg.

[23] Bangladesh Bank. Exchange rate of taka. Available: http://www.bangladesh-bank.org/econdata/exchangerate.php. Accessed: 28 January 2011.

[24] Bangladesh Bureau of Statistics. Population census–2001, community series. 2001. Bangladesh Bureau of Statistics: Dhaka: Bangladesh. Available: http://www.bbs.gov.bd. Accessed: 28 June 2013.

[25] Molinari NA, Ortega-Sanchez IR, Messonnier ML, Thompson WW, Wortley PM, Weintraub E (2007). The annual impact of seasonal influenza in the US: measuring disease burden and costs.. Vaccine.

[26] Xue Y, Kristiansen IS, de Blasio BF (2010). Modeling the cost of influenza: the impact of missing costs of unreported complications and sick leave.. BMC Public Health.

[27] Vyas S, Kumaranayake L (2006). Constructing socio-economic status indices: how to use principal components analysis.. Health Policy Plan.

[28] National Institute of Population Research and Training (NIPORT). Mitra and Associates, and Macro International 2009. Bangladesh Demographic and Health Survey 2007. Dhaka, Bangladesh and Calverton, Maryland, USA: National Institute of Population Research and Training (NIPORT), Mitra and Associates, and Macro International. Available: http://www.niport.gov.bd/document/research/BDHS-2007-Final-Report.pdf. Accessed: 16 August 2011.

[29] Duggal R (2007). Poverty & health: criticality of public financing.. Indian J Med Res.

[30] World Health Organization. Health System in Bangladesh. Available: http://www.whoban.org/en/Section25.htm. Accessed: 19 December 2011.

[31] McIntyre D, Thiede M, Dahlgren G, Whitehead M (2006). What are the economic consequences for households of illness and of paying for health care in low- and middle-income country contexts?. Soc Sci Med.

[32] Institute of Microfinance. Interest Rates in Bangladesh Microcredit Market. Available: http://www.inm.org.bd/publication/briefs/Interest%20Rate.pdf. Accessed: 2 May 2014.

[33] van Doorslaer E, O’Donnell O, Rannan-Eliya RP, Somanathan A, Adhikari SR, Garg CC (2007). Catastrophic payments for health care in Asia.. Health Econ.

[34] van Doorslaer E, O’Donnell O, Rannan-Eliya RP, Somanathan A, Adhikari SR, Garg CC (2006). Effect of payments for health care on poverty estimates in 11 countries in Asia: an analysis of household survey data.. Lancet.

[35] Su TT, Kouyate B, Flessa S (2006). Catastrophic household expenditure for health care in a low-income society: a study from Nouna District, Burkina Faso.. Bull World Health Organ.

[36] Mondal S, Kanjilal B, Peters DH, Lucas H. Catastrophic out-of-pocket payment for health care and its impact on households: experience from West Bengal, India. 2010. Available: http://www.chronicpoverty.org/uploads/publication_files/mondal_et_al_health.pdf. Accessed: 2 June 2014.

[37] Ahmed T, Ahmed AMS (2009). Reducing the burden of malnutrition in Bangladesh.. BMJ.

[38] Bangladesh Bureau of Statistics. Report of the household income and expenditure survey 2010. 2011. Bangladesh Bureau of Statistics: Dhaka, Bangladesh. Available: http://www.bbs.gov.bd. Accessed: 15 February 2015.

[39] Ranson MK, Sinha T, Chatterjee M, Acharya A, Bhavsar A, Morris SS (2006). Making health insurance work for the poor: learning from the Self-Employed Women’s Association’s (SEWA) community-based health insurance scheme in India.. Soc Sci Med.

[40] Isaacs D, Dickson H, O’Callaghan C, Sheaves R, Winter A, Moxon ER (1991). Handwashing and cohorting in prevention of hospital acquired infections with respiratory syncytial virus.. Arch Dis Child.

